# Antibacterial Biofilms of Chitosan Incorporated with the Ethanolic Extract of the Stem Bark of *Libidibia ferrea* and Its Fractions

**DOI:** 10.3390/molecules31091392

**Published:** 2026-04-23

**Authors:** Andreza Santos de Jesus, Aiane Nascimento Santana, Helena Carla Magalhães dos Reis, Giovanna Regina Gonzalez de Santana Wojnar, Vitor Hugo Migues, Arnaud Victor dos Santos, Madson de Godoi Pereira, Lourdes Cardoso de Souza Neta, Sandra Aparecida Alexandre Lucas, Rodrigo Lassarote Lavall

**Affiliations:** 1 Department of Exact and Earth Sciences I, UNEB-State University of Bahia, Silveira Martins Street, 2555 Cabula, Salvador 41150-000, Bahia, Brazil; dreza.santos@hotmail.com (A.S.d.J.); aianesantana92@gmail.com (A.N.S.); hcarla01.hc@gmail.com (H.C.M.d.R.); giovannawojnar@gmail.com (G.R.G.d.S.W.); vhmigues@gmail.com (V.H.M.); arnaudvic@gmail.com (A.V.d.S.); mpereira@uneb.br (M.d.G.P.); 2 Department of Chemistry, Institute of Exact Sciences, UFMG-Federal University of Minas Gerais, Antônio Carlos Av., 6627 Pampulha, Belo Horizonte 31270-901, Minas Gerais, Brazil; salexandre2007@gmail.com (S.A.A.L.); rodrigo.lavall@qui.ufmg.br (R.L.L.)

**Keywords:** antibacterial, biomaterial, films, Fabaceae

## Abstract

The high mortality rate from microbial infections underscores the need to discover new antimicrobials. This work produced antibacterial Chitosan biofilms with and without the incorporation of the ethanolic extract of *Libidibia ferrea* stem bark and its ethyl acetate and aqueous fractions. The extract and fractions were subjected to FTIR and ^1^H NMR analysis. The biofilms were characterized by FTIR, scanning electron microscopy, thermogravimetry, and differential scanning calorimetry analysis. The ^1^H NMR and FTIR data, as well as the colorimetric quantification of total phenolics, demonstrated the presence of phenolic compounds. *Staphylococcus aureus* and *Bacillus cereus* were the most susceptible bacteria for Chitosan/*L. ferrea* biofilms and fractions (growth inhibition zones values in the range of 10.8 ± 0.1 to 14.0 ± 0.1 mm, and minimum inhibitory or bactericidal concentration, MIC or MBC values of the fractions were in the range of 125 to 250 µg mL^−1^. Only the fractions inhibited *Pseudomonas aeruginosa* (MIC = 250 µg mL^−1^). Chitosan/*L. ferrea* biofilms exhibited efficient interactions between chitosan functional groups and secondary metabolites, good thermal stability, and increased rigidity in mechanical tests. This study reinforces the pharmacological potential of biodegradable Chitosan/*L. ferrea* biofilms as antibacterial agents biofilms.

## 1. Introduction

The increase in mortality due to bacterial infections, especially due to antimicrobial resistance, is one of the most serious global public health problems, according to the World Health Organization (WHO) [1,2,3]. Among bacteria, Gram-positive bacteria, such as those in the genus *Staphylococcus*, can cause infections in immunocompromised individuals, leading to skin and respiratory diseases (such as pneumonia and acute bronchitis), sepsis, and endocarditis [4]. Additionally, other Gram-positive bacteria present risks; for example, *Bacillus cereus* causes food poisoning and, consequently, gastroenteritis, vomiting, or diarrhea [5].

*Pseudomonas aeruginosa*, a Gram-negative bacterium identified by the WHO as a serious public health threat, causes severe lung infections [6,7]. As a result, treating these infections is challenging due to the rise in multidrug-resistant and conventional antimicrobial-resistant strains [1,2,3,8]. Antimicrobial resistance (AMR) occurs when microorganisms become resistant to antibiotics through intrinsic or acquired mechanisms, limiting treatment options for infections [9,10]. The WHO estimates that by 2050, such microorganisms could cause over 10 million deaths, underscoring AMR’s critical public health relevance. This situation highlights the urgent need to discover and develop new antimicrobial compounds [11].

The traditional use of natural products in treating and preventing diseases has long contributed to the development of new medicines, particularly by enabling the discovery of secondary metabolites effective against various diseases, such as bacterial infections [12,13]. Isolated plant compounds, including flavonoids, alkaloids, polyphenols, terpenoids, quinones, saponins, and lactones, have shown efficacy against diverse biological targets [13,14,15]. Examples such as anthocyanins, naringenin, ellagic acid, and catechins demonstrate antimicrobial activity against various microorganisms [16,17,18,19,20,21].

In addition to these secondary metabolites, researchers have sought new antibacterial and antifungal strategies as alternatives to commercial antibiotics. Among these alternatives are inorganic nanomaterials, including metallic and metal oxide nanoparticles such as silver nanoparticles [22], as well as antibacterial fabrics [23]. Moreover, methods such as photodynamic therapy [24] have been explored. In addition, other promising approaches include organic–inorganic hybrid materials like organocyclophosphazenes [25,26], antimicrobial peptides [27], carbon nanomaterials [28], carbon-based quantum dots [29], and natural biopolymer films with medicinal plant extracts [30].

Chitosan is a promising matrix for antimicrobial films because it is a biodegradable and non-toxic copolymer. It is derived from the deacetylation of chitin, a polysaccharide, and exhibits antimicrobial activity against many bacteria and fungi [31,32]. These activities may relate to molecular weight, degree of deacetylation, or concentration. The origin and polycationic nature of chitosan may also contribute to its antibacterial activity. Its polycationic character enables interactions with bacterial membranes and biomolecules such as DNA and RNA [33,34,35].

Given the antimicrobial properties of chitosan and secondary metabolites, the association of plant extracts and their fractions with natural polymers has emerged as a strategy for preparing biodegradable biomaterials with improved antimicrobial efficacy and/or potential for use in packaging applications or as dressings [36,37]. Therefore, biodegradable chitosan biopolymers were prepared by incorporating various plant extracts, such as pink pepper, thyme, beet leaves, rosemary, fruit extracts, and cinnamon barks, among others [38,39,40,41,42,43,44,45,46]. In this context, antimicrobial biomaterials made from chitosan and natural sources have diverse potential uses in general merchandise and biodegradable food packaging. Specifically, these materials can replace plastic packaging (petrochemical-based), which harms ecosystems due to its persistence [47,48]. Furthermore, they also offer promising options for wound dressings [49].

*Libidibia ferrea* (MART. Ex TUL.) L.P. QUEIROZ (synonym *Caesalpinia ferrea*, genus *Libidibia*; family Fabaceae) is a promising option for producing biologically active biomaterials. Traditionally, it is used as an antimicrobial and anti-inflammatory agent in the treatment of bronchitis, infections, diabetes, and wounds [50,51,52,53,54,55,56]. Moreover, extracts and fractions of *L. ferrea* have demonstrated diverse biological activities, including antibacterial, anticancer, cytotoxic, analgesic, antioxidant, wound-healing, anti-inflammatory, and anti-ulcer properties [53,54,56,57,58,59,60,61,62]. The classes of metabolites identified in *L*. *ferrea* extracts were catechin, quercetin, ellagic acid, gallic acid, apigenin, kaempferol, rutin, linoleic acid, steroids, condensed tannins, hydrolyzable tannins, saponins, coumarins, among others [19,63,64,65,66,67,68,69].

Regarding the antimicrobial activity of *L. ferrea*, fruit, stem and leaves extracts showed activity against *Staphylococcus aureus*, *Streptococcus mutans*, *S. oralis*, *S. salivarius*, *Lactobacillus casei*, and *Candida albicans* [54,70,71]. Stem extracts showed activity against *S. aureus*, *S. epidermidis*, *Bacillus subtilis*, *B. cereus*, *P. aeruginosa*, *E. coli*, *Cronobacter* sp., *Salmonella* sp., and *C. albicans* [68,72,73,74,75].

In this context, biodegradable chitosan biofilms were produced with and without the incorporation of the ethanolic extract of *L. ferrea* stem bark, as well as its ethyl acetate and aqueous fractions. Additionally, the antibacterial and antifungal potential of the *L. ferrea* extract, its fractions, and chitosan and chitosan/*L. ferrea* biofilms were evaluated against bacterial strains (Gram-positive and Gram-negative) and against the yeast *Candida albicans*.

## 2. Results

### 2.1. Results of Spectroscopic Analyses of L. ferrea Samples

#### 2.1.1. H NMR Data of the Ethanolic Extract of *L. ferrea* Stem Bark and Its Fractions

The ^1^H NMR spectrum of the ethanolic extract of *L. ferrea* stem bark (ELF) showed mainly signals in the aromatic hydrogen region at δ_H_ 6.00 to δ_H_ 8.00. In addition to signals at δ_H_ 3.47 to δ_H_ 5.39 (Figure S1a, Supplementary Data). The signals in the ^1^H NMR spectra of the ethyl acetate fraction of the ethanolic extract of the stem bark of L. ferrea (ELFA) (Figure S1b, Supplementary Data) and the aqueous fraction of the ethanolic extract of the stem bark of *L. ferrea* (ELFAq) (Figure S1c, Supplementary Data) were similar to those of ELF; however, the signals in the δ_H_ 7.06 to δ_H_ 6.20 and δ_H_ 5.35 to δ_H_ 4.03 regions were more intense in the ^1^H NMR spectrum of fraction ELFA than in the spectra of extract ELF and fraction ELFAq.

#### 2.1.2. FTIR Data of the Ethanolic Extract of *L. ferrea* Stem Bark and Its Fractions

**Figure 1:**
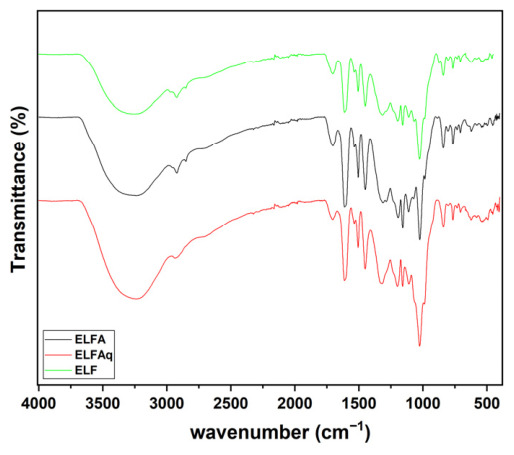
FT-IR spectra of the ethanolic extract of *L. ferrea* stem bark (ELF) and its ethyl acetate (ELFA) and aqueous (ELFAq) fractions.

The infrared absorption spectra of the ethanolic extract of *L. ferrea* stem bark (ELF) and its aqueous fraction of the ethanolic extract of the stem bark (ELFAq) and ethyl acetate fraction of the ethanolic extract of the stem bark (ELFA) are shown in Figure 1.

### 2.2. Total Phenolics

Total phenolic values, measured by the Folin–Ciocalteu method, were 160.95 ± 0.95 mg GAE g^−1^ for ELFA and 97.05 ± 2.15 mg GAE g^−1^ for ELFAq (mg gallic acid equivalent per g of fraction; y = 0.048x − 0.00431, R2 = 0.9972).

### 2.3. Characterization of Chitosan Biofilms with and Without L. ferrea Samples

#### 2.3.1. FT-IR Spectra Analysis of Biofilms

**Figure 2:**
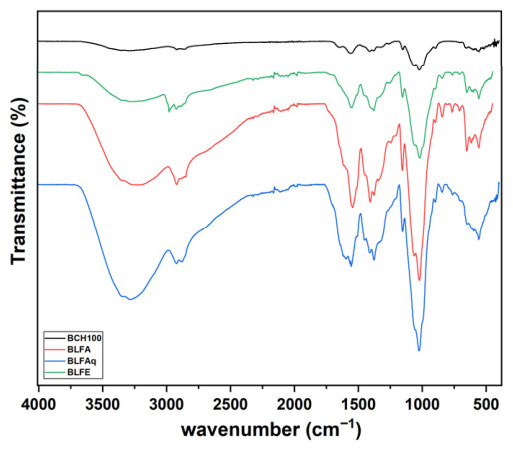
FT-IR spectra of chitosan biofilms: BCH100 (pure chitosan biofilm), BLFE (chitosan biofilm incorporating ethanolic extract of *L. ferrea* stem bark), BLFA (chitosan biofilm with ethyl acetate fraction) and BLFAq (chitosan biofilm with aqueous fraction).

The infrared absorption spectra of the biofilm without *L. ferrea* samples (BCH100) and with incorporation of the ethanol extract of *L. ferrea* stem bark (BLFE) and its ethyl acetate (BLFA) and aqueous (BLFAq) fractions showed similarities in spectral features, however, with differences in intensities and band broadening (Figure 2).

#### 2.3.2. Thermal Properties of Biofilms

**Figure 3:**
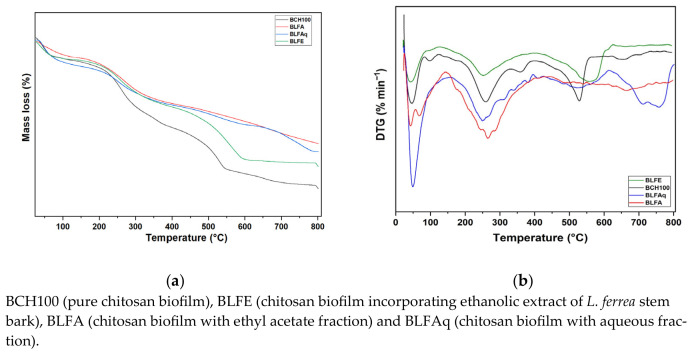
TGA and DTG curves of chitosan biofilms with and without incorporation of *L. ferrea* samples: (**a**) TGA curve; (**b**) DTG curve.

Thermogravimetric analyses of the BCH100, BLFE, BLFA, and BLFAF biofilms were performed to assess their thermal stability. The thermogravimetry and derivative thermogravimetry (TG/DTG) curves showed three stages of mass loss of the biofilms (Figure 3 and Table 1).

In the first stage, the BCH100, BLFE, BLFA, and BLFAq biofilms showed mass losses in the following temperature ranges: 39.26 °C to 59.63 °C (14.467% loss); 37.78 °C to 63.76 °C (14.500% loss); 40.71 °C to 74.55 °C (12.009% loss); and 45.39 °C to 73.07 °C (19.731%) (Table 1). In the second stage of biofilm degradation, the approximate temperature range was 200.0 °C to 311.0 °C [BCH100 (230.43 °C to 287.76 °C); BLFE (220.20 °C to 296.67 °C); BLFA (208.74 °C to 310.20 °C) and BLFAq (224.40 °C to 300.98 °C), resulting in a mass loss of 42.437%; 35.152%; 37.17% and 33.379%, respectively. The third mass loss for BCH100 occurred in the range of 504.06 °C to 541.73 °C, with a loss of 34.365%. The BLFE, BLFA, and BLFAq biofilms showed losses of 40.777, 34.984, and 41.467, respectively, in ranges varying from 510.84 to 780.00 °C (Table 1).

#### 2.3.3. Appearance and Surface Analysis of Biofilms

**Figure 4:**
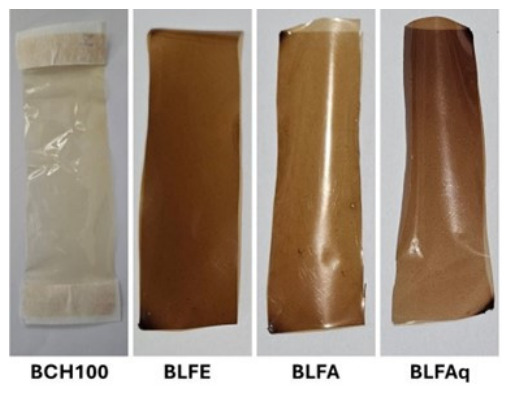
Appearance of the biofilms: BCH100 (pure chitosan biofilm), BLFE (chitosan biofilm incorporating ethanolic extract of *L. ferrea* stem bark), BLFA (chitosan biofilm with ethyl acetate fraction) and BLFAq (chitosan biofilm with aqueous fraction).

The appearance of chitosan biofilms with and without incorporation of *L. ferrea* samples is shown in Figure 4.

**Figure 5:**
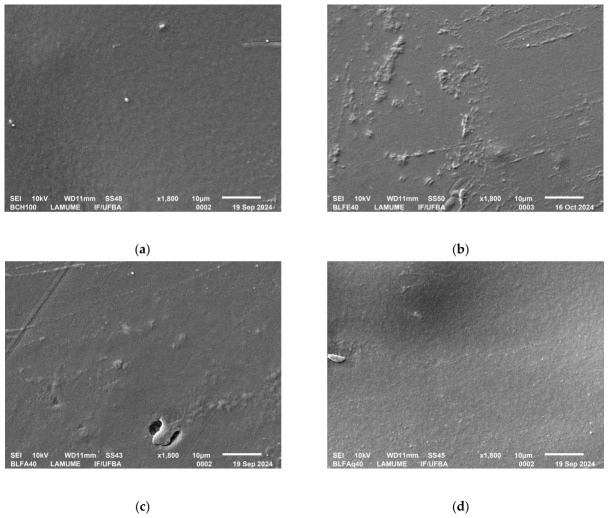
Micrographs of chitosan biofilms with and without incorporation of *L. ferrea* biofilm. of chitosan biofilms: (**a**) BCH100 (Pure chitosan biofilm); (**b**) BLFE (Chitosan biofilm incorporating ethanolic extract of *L. ferrea* stem bark); (**c**) BLFA (Chitosan biofilm with ethyl acetate fraction) and (**d**) BLFAq (Chitosan biofilm with aqueous fraction).

Micrographs of the biofilms obtained by scanning electron microscopy are shown in Figure 5. Specifically, the BCH100 biofilm displays a more homogeneous surface with low porosity and few aggregates (Figure 5a) compared to the BLFE (Figure 5b) and BLFA (Figure 5c) biofilms. In contrast, BLFE and BLFA (Figure 5b,c) biofilms showed greater surface roughness and aggregates. Notably, the BLFAq biofilm (Figure 5d) showed greater morphological similarity to BCH100. Furthermore, analysis of the energy-dispersive spectroscopy spectra of the BCH100, BLFAq, and BLFA biofilms showed that the main elements present in all films were carbon and oxygen (Figure S2, Supplementary Data).

#### 2.3.4. Thickness and Mechanical Properties of Biofilms

The thickness measurements and mechanical parameters, such as tensile strength and stress, of biofilms are presented in Table 2. The data on thickness showed that the BLFE, BLFAq, and BLFA biofilms have significantly greater thickness than the BCH100 chitosan biofilm at the 95% confidence level (two-sample *t*-test). Regarding the breaking strength (burst strength) of the biofilms, the BCH100 biofilm showed an average of 17.50 ± 14.81 N. In comparison, the chitosan biofilms incorporating ethyl acetate and aqueous fractions showed average values of 39.83 ± 13.22 N and 40.92 ± 11.30 N, respectively. The BLFAq biofilm showed the highest tensile strength (40.92 N), indicating greater uniformity and structural resistance among the formulations tested (Table 2).

#### 2.3.5. Biodegradability Test in Soil and Water of the Biofilms

Biofilms of pure chitosan and chitosan with incorporated fractions were subjected to biodegradation tests in soil and water. After 25 days, the percentage of mass loss was obtained in these two environments (Table 3).

### 2.4. Antimicrobial Activity

All samples tested (ELF extract, ELFA and ELFAq fractions, BCH100 (pure chitosan biofilm) BLFE, BLFA, and BLFAq biofilms) using the agar diffusion method were selective for the Gram-positive bacteria *Staphylococcus aureus* and *Bacillus cereus*. Under the tested conditions, no sample inhibited fungal growth (Table 4). There was a significant difference in the inhibition zones (IZ) between chitosan biofilms produced with the incorporation of the aqueous fraction (BFLAq) and the ethyl acetate fraction (BLFA), and the respective inhibition zones of the aqueous and ethyl acetate fractions against *S. aureus* (95% confidence using the *t*-test for comparing two means). However, for *B. cereus*, there was no statistically significant difference between the inhibition zones of the BLFAq and BLFA biofilms and their respective ELFAq and LFA fractions (95% confidence using the *t*-test for comparing two means). The chitosan biofilm without incorporation of the ethanolic extract of *L. ferrea* stem bark (BCH100, pure chitosan) showed only a growth inhibition zone for *B. cereus* (IZ = 13.6 ± 0.1 mm), among all microorganisms tested. The ethanolic extract of the leaves (ELF) inhibited *B. cereus* (IZ = 14.0 ± 0.1 mm), while the biofilm prepared with this extract did not show antibacterial activity against *B. cereus*. This suggests that the compounds present in the extract and in the biofilm with chitosan had an antagonistic effect against *B. cereus* (Table 4).

In the broth microdilution assay, both the ethyl acetate and aqueous fractions of the ethanolic extract of *L. ferrea* stem bark showed antibacterial activity against *S. aureus* and *B. cereus*, Gram-positive bacteria, and *P. aeruginosa*, Gram-negative bacteria, with minimum inhibitory concentration (MIC) values in the range of 500 to 125 µg mL^−1^. Among these, the acetate fraction was the most active against Gram-positive bacteria (MIC = 125 µg mL^−1^) (Table 5).

## 3. Discussion

^1^H NMR data from the ELF extract and the ELFa and ELFAq fractions suggest the presence of aromatic acids derived from phenolic compounds, such as gallic and ellagic acid derivatives [76,77,78,79], and hydrolyzable tannins [80] (Figure S1, Supplementary Data). Signals in the *δH* 3.47–5.39 region indicate hydrogens on sugar skeletons characteristic of glycosylated flavonoids (Figure S1a, Supplementary Data) [79,81,82]. These findings align with previous phytochemical studies of the genus *Libidibia*, which identified phenolic compounds in these species [78,83,84,85,86,87].

The infrared absorption spectra of the ELF extract and its ELFA and ELFAq fractions (Figure 1) display a broad band in the range of 2500 cm^−1^ to 3500 cm^−1^, with the highest intensity at 3239 cm^−1^ due to O-H bond stretching. In addition, other overlapping bands appear at 1700–1650 cm^−1^ in the spectra, corresponding to the stretching of the C=O bond (carbonyl functional group). Angular vibrations at 1454 cm^−1^ and in the stretching range from 1322 cm^−1^ to 1022 cm^−1^ (overlapping) of C-H and C-O bonds, respectively, can also reinforce the presence of alcohols, ethers, phenolic compounds, and polysaccharides polissacarídeos [88,89].

These findings indicate the presence of phenolic compounds in the extract and fractions, including carboxylic acid derivatives, glycosylated flavonoids, and tannins, as observed in the ^1^H NMR data [72,78,89,90,91]. The determination of the total phenolic in the ELFA and ELFAq fractions corroborates the ^1^H NMR data of these fractions and is in agreement with the data obtained in the literature for the ethanolic extract of the stem bark of *L. ferrea* [68]. Previous studies with *L. ferrea* have quantified phenolic compounds, including catechin, gallic acid, epicatechin, and ellagic acid, in extracts of its stem and aerial parts [85,92]. Additionally, studies show that the total phenolic content of the hydromethanolic extract of *L. ferrea* stems was 0.151 ± 0.005 mg GAE g^−1^, determined by the Folin–Ciocalteu method [93]. De Araújo et al. (2014) [84] obtained the flavonoid content and total phenolic of the aerial part extract of *L. ferrea*, using spectrophotometric methods, of 4.56 ± 1.58 mg g^−1^ GAE and 68.13 ± 15.93 mg g^−1^, respectively.

In all FT-IR spectra of the biofilms, a broad band was observed in the 3400–2600 cm^−1^ region. This band covered the entire spectrum and had a maximum at 3286 cm^−1^. The overlap of the stretching vibrations of O-H and N-H bonds is indicated (Figure 2). These stretching patterns suggest the presence of amino and hydroxyl groups. These groups originate from chitosan alcohols (about 3000–3400 cm^−1^) and from phenolic acids and flavonoids in the chitosan/extract and chitosan/fractions biofilms [94]. FTIR spectra show greater intensity and broader bands in chitosan biofilms with the incorporation of the extract or fraction than in those without *L. ferrea* samples (Figure 1) [46,88,89,91,95,96].

The presence of phenolic compounds in the extract and its fractions, as observed in the ^1^H NMR and FTIR data of the extract and fractions, may contribute to the increased intensity of these bands. In all infrared spectra of chitosan biofilms (100%) and with the incorporation of *L. ferrea* samples, the overlap of absorption bands at 1557 cm^−1^ (range of 1500–1600 cm^−1^) is in the region of N-H angular vibrations of the amino group, which, together with the absorption bands in the range of 1025 cm^−1^ to 1322 cm^−1^, characterize the presence of stretching vibrations of the C-OH, C-O-C and C-C-O bonds and C-N angular vibrations, confirming the presence of glycopyranosidic derivatives of chitosan [32,96,97].

The FT-IR spectra of chitosan biofilms with *L. ferrea* samples displayed characteristic aromatic C-C bond vibration bands at 1605 cm^−1^, 1504 cm^−1^, and 1459 cm^−1^ [89,94], also present in the FTIR spectra of *L. ferrea* extract and fractions. Additionally, the bands between 900 cm^−1^ and 700 cm^−1^ (with peaks at 844 cm^−1^, 767 cm^−1^, and 710 cm^−1^) indicate angular deformations of C-O-C bonds in glycosidic links and C-H bonds in aromatic compounds [96,97,98]. TG/DTG curves were used to evaluate the thermal stability of chitosan biofilms without (BCH100) and with incorporation of the ethanolic extract of *L. ferrea* stem bark and its fractions in ethyl acetate and aqueous fraction (BLFE, BLFA, and BLFAq) (Figure 3).

The initial mass loss in biofilms likely results from evaporation of water and volatile solvents at 30 °C to 110 °C [99]. A second stage of mass loss is attributed to the decomposition of glycerol. Huong et al. (2026) [95] reported that chitosan present in *Pseuderanthemum palatiferum* leaf extract causes mass loss above 50% between 150 °C and 300 °C. Furthermore, the onset of chitosan decomposition occurs in the temperature range of 210 °C to 300 °C [99,100,101]. At temperatures above 400 °C, the losses are attributed to the complete depolymerization of the polysaccharides, including the breakage of the pyran ring and the cleavage of the glycosidic bonds between glucosamine and N-acetylglucosamine [97,102]. Furthermore, this loss may be due to the oxidative degradation of carbonaceous residues and aromatic rings present in the plant material, as obtained by Fajardo et al. (2025) [97] for the methanolic extract of *Acmella oleracea* leaves, with mass loss in the range of 670 to 730 °C. In the first stage, it was observed that incorporating the ethyl acetate and aqueous fractions into chitosan biofilms resulted in a slight increase in the temperature range, with little impact on mass loss. However, in the third stage, the incorporation of these fractions and the extract into chitosan biofilms increased the degradation range of the biofilms (BLFE, BLFA, and BLFAq), in agreement with the findings of Fajardo et al. (2025) [97]. Studying thermal stability allows us to determine the maximum temperature at which the film can be processed without inactivating the active component. Therefore, even when the main focus is on antimicrobial activity, thermal stability is fundamental because temperature directly affects the material’s structure and the effectiveness of the active agents incorporated into it. Thermal stability ensures the film performs consistently in real-world applications, such as food packaging and medical devices.

Chitosan biofilms incorporating ethanolic extract of *L. ferrea* stem bark (BLFE) and its ethyl acetate (BLFA) and aqueous fractions showed a brown coloration, which was visually darker in the BLFE and BLFAq (BLFAq) biofilms compared to the chitosan biofilm without incorporation of *L. ferrea* samples (BCH100) (Figure 4).

In SEM analyses, chitosan biofilms incorporating *L. ferrea* samples showed decreased homogeneity compared to chitosan biofilms without plant material incorporation (Figure 5). This finding is in agreement with that obtained in the literature [102,103] in chitosan films prepared with the hydroalcoholic extract of jaboticaba bagasse (*Jaboticaba pomace*) (concentrations used of the extract: 2.5; 5; 10 and 20 m m^−1^) and by Fajardo et al. (2025) [97] in chitosan films prepared with the methanolic extract of jambu leaves (*A. oleracea*) (concentrations used of the extract: 5.0%, 2.5% and 1.0% m m^−1^), among others [46,96,100]. The greater roughness of the BLFE (Figure 5b) and BLFA (Figure 5c) biofilms suggests a higher concentration of insoluble compounds, such as fats, in the chitosan matrix during film preparation [95].

The BLFAq biofilm (Figure 5d) showed porosity, with some holes on the surface and maintenance of a few surface reliefs, compared to the BLFE and BLFA biofilms, with an appearance similar to white (BCH100), suggesting a greater compatibility of the constituents of the aqueous fraction with the chitosan matrix, consistent with the micrograph found by Iquiapaza et al. (2025) [96] in chitosan biofilms with aqueous propolis extract. Among all the films, BLFAq showed the greatest potential for application as a wound dressing, as its surface is most similar to that of the pure chitosan film (BCH100). The compatibility between the secondary metabolites and the chitosan matrix can be improved by optimizing the amounts of extracts added to the matrix, as obtained by incorporating the ethanolic extract of *Sonneratia caseolaris* (L.) Engl. leaves into the chitosan matrix [104].

The element nitrogen was not detected on the surfaces, as expected from the chitosan structure, suggesting that nitrogen atoms may be arranged towards the interior of the matrix in biofilms. In this study, carbon, oxygen, and sodium were also identified as the main components of chitosan–alginate–neomycin–lidocaine hybrid biofilms [105].

Regarding thickness, BLFE was the thickest of all, which may be due to the presence of soluble solids and/or phenolic compounds in the extract, among other factors [46]. This behavior was also observed in chitosan-pectin films incorporated with the ethanolic extract of *Piper betle* L. leaves [106], methylcellulose films and the aqueous extract of jambolan bark (*Syzygium cumini*) [36], and chitosan with extracts of purslane, *Portulaca oleracea* L. [107].

The tension obtained for the BCH100 biofilm was 28.62 ± 15.31 Mpa (Table 2), within the range reported in the literature [106,107,108,109]. This value was lower than that found for biofilms prepared by incorporating *L. ferrea* samples (BLFE, BLFA, and BLFAq). These results are consistent with preliminary studies, as in films prepared with methylcellulose and the aqueous extract of jambolan bark (*Syzygium cumini*) [45], and in sodium alginate films with the hydroalcoholic extract of guava leaves, *Psidium guajava* Linn [100]. However, this result was contrary to that obtained by Fan et al. (2023) [107], who observed a decrease in the stiffness of chitosan films with *P. oleraceae* L. extract.

Regarding the biofilm rupture strength, there was a statistically significant difference between BCH100 and the biofilms produced with *L. ferrea* samples: BLFE, BLFAq, and BLFA. However, among these, there was no significant difference at the 95% confidence level (*t*-test for two means). The compounds present in the extract and fractions acted as mechanical enhancers, favoring intermolecular interactions between the chitosan chains, such as hydrogen bonds and hydrophobic interactions, resulting in a more cohesive and resistant structure.

The biodegradation of the films in water and soil was expressed as percent weight loss. Chitosan biofilms with and without *L. ferrea* incorporation showed no significant difference in biodegradation in soil and water (95% confidence, *t*-test for two means). There was a significant difference in biodegradation in an aquatic environment between BCH100 and biofilms of the following fractions: BFA and BLFAq. In soil, there was a significant difference between BCH100 and BLFAq. However, there was no significant difference between BCH100 and BLFA (95% confidence using the *t*-test for comparing two means). These findings suggest that adding fractions to chitosan biofilms delayed microbial biodegradation, primarily of the chitosan glycosidic bonds, in soil [110,111].

In the biodegradation test in water, similarly, there was also a greater loss of mass in the pure chitosan film (BCH100), compared to the others. Degradation in an aqueous environment is generally influenced by the material’s solubility and swelling, factors that are relevant for pure chitosan and thus facilitate microbial attack [112]. Soil moisture readily penetrates the polymer network, weakens the polymer chains, and makes them susceptible to hydrolysis by soil microbes at the glycosidic bonds.

The ethanol extract of *L. ferrea* stem bark and its ethyl acetate and aqueous fractions, along with chitosan biofilms with and without *L. ferrea* (BCH100, pure chitosan) samples showed antibacterial activity using both methods. In this study, all samples tested were selective for the Gram-positive strains *S. aureus* and *B. cereus* by the agar diffusion method, while, by the broth dilution method, the ELFAq and ELFA fractions from the ELF extract were also active against *S. aureus*, *B. cereus*, and, additionally, against the Gram-negative *P. aeruginosa*.

In a previous seasonal study, the ELF extract, which was collected in the spring season in the Atlantic Forest biome (Bahia, Brazil), showed selectivity for Gram-positive strains, such as *S. aureus*, *B. subtilis*, *B. cereus*, and *Staphylococcus epidermidis* (MICs = range of 62.5 to 500 µg mL^−1^). In addition, ELF showed a higher total phenolic content, with a significant difference observed only in the summer season. The highest flavonoid content was observed in the winter and autumn seasons, at the 98% confidence level (Tukey test) [68]. Extracts and fractions of *L. ferrea* have already demonstrated antibacterial effects against *S. aureus.* Specifically, alcoholic extracts (including ethanol) from both leaf and stem bark exhibited MICs ranging from 100 to 0.39 µg mL^−1^ [73]; the hydroalcoholic fraction of the stem bark showed an MIC of 0.39 mg mL^−1^ [72]; and the ethanolic extract of the leaf had an MIC of 250 μg mL^−1^ [113]. The ethanolic extract of *L. ferrea* pods inhibited *Enterococcus faecalis*, *B. subtilis*, *S. aureus*, *E. coli*, *K. pneumoniae*, and *P. aeruginosa*. The MIC values ranged from 50 to 125 µg mL^−1^ [114]. Santana et al. (2024) [68] evaluated the antimicrobial activity of *L. ferrea* extracts from the Brazilian biome using the microdilution method. They found that ethanolic extracts of *L. ferrea* stem bark, collected in winter from the Atlantic Forest biome, were effective against *S. epidermidis* (MIC = 125.0 μg mL^−1^; MBC = 500.0 μg mL^−1^). The Caatinga biome stem extract, though more selective for Gram-positive bacteria, also inhibited *P. aeruginosa* and *E. coli*, in all seasons of the year, with MICs of 500 μg mL^−1^ and MBCs of 2000 μg mL^−1^ [68].

In the agar diffusion assay, there was a significant difference between the zones of microbial growth inhibition of *S. aureus*, with the fractions being more active than their respective biofilms. However, the pure chitosan film (BCH100) showed no effect against this bacterium under the experimental conditions employed. Given this result, there was little suppression of the groups in the active principles and potential pharmacophores in the BLFAq and BLFA chitosan biofilms. Meanwhile, for B. cereus, BCH100 showed activity, and the incorporation of the fractions into the chitosan matrix showed an antagonistic effect.

The greater antibacterial activity demonstrated by *L. ferrea* fractions against *S. aureus* and *B. cereus*, and by its chitosan biofilms against *S. aureus*, can be attributed to the presence of phenolic compounds, which are known to exert antibacterial effects. These compounds are reported to be responsible for the biological activities of *L. ferrea*, among others [54]. The phenolic compounds and flavonoids present in *L. ferrea* can act through various mechanisms, including altering bacterial membrane permeability, leading to loss of cellular integrity [115], inhibiting intracellular enzymes and the respiratory chain [116], and binding to proteins and bacterial DNA, thereby compromising essential metabolic processes. These mechanisms explain the greater effectiveness against Gram-positive bactéria [117,118,119]. Although the aqueous fraction (ELFAq) had lower phenolic content than the ethyl acetate fraction (ELFA), the chitosan biofilm (BLFAq) with ELFAq showed better homogeneity and tensile strength. This suggests more effective intermolecular interactions between the chitosan matrix and phenolic compounds in the aqueous fraction. The antibacterial effect of the biofilm fractions suggests that the active principles diffused more from BLFAq into the nutrient agar. This is shown by the similar microbial growth inhibition zones against *S. aureus* and *B. cereus* (95% confidence, *t*-test for two means).

The bacteria have cell walls composed of peptidoglycan, a linear polysaccharide chain linked by tetrapeptides, which provide protection and support. Specifically, Gram-positive bacteria have thicker walls and an inner membrane, whereas Gram-negative bacteria have an outer membrane, a thinner cell wall, and an inner membrane. The outer membrane of Gram-negative bacteria, rich in lipopolysaccharides, acts as a barrier against the action of antibiotics, especially those that affect peptidoglycan synthesis, such as penicillin [53,114,120]. All samples tested were inactive against *C. albicans*. Further assays with more fungal strains are needed to determine selectivity [121].

The results obtained against these bacteria are relevant given the increasing mortality from infections caused by antibiotic-resistant bacteria, especially among immunocompromised patients. Notably, according to the WHO, *P. aeruginosa* and *S. aureus* are among the priority pathogens. Furthermore, the observed effect on *B. cereus* may contribute to the long-term treatment of gastrointestinal infections. Taken together, these results demonstrate the potential of the *L. ferrea* stem extract and its fractions to provide antibacterial compounds, as evidenced by bio-guided phytochemical studies on this activity [4,6,7].

Chitosan offers beneficial properties, including biodegradability, air permeability, biocompatibility, low cytotoxicity, analgesic effects, skin repair, and antimicrobial activity [104,122]. Antibacterial chitosan biofilms prepared from the ethanolic extract of *L. ferrea* stem bark and its fractions have shown promise for long-term biomedical use. These biofilms are effective mainly against *S. aureus* and *B. cereus*. They may serve as dressings for infectious wounds or as antimicrobial food packaging, including active packaging to prevent food contamination. Among all biofilms, the biofilm produced with the incorporation of the aqueous fraction of the *L. ferrea* etanolic extract was the most promising for future use as an antibacterial dressing, due to its mechanical properties and good homogeneity, both of which meet the requirements sought in dressings [104,122].

The possibility of using chitosan/*L. ferrea* biofilms as dressings are also promising when compared to those made with other plant extracts [38,39,40,41,42,43,44,45,46], since the results obtained support the medicinal use of *L. ferrea*. Ointments produced with *L. ferrea* bark powder demonstrated healing capacity in cutaneous wounds with a reduction in bacterial infection caused by *S. aureus* [93]. Given the findings of this study, antibacterial biofilms produced with *L. ferrea* samples, particularly the aqueous fraction, contribute to the medicinal use of this species in the treatment of infections and reinforce the potential application of these biofilms as dressings. However, to achieve these applications, chitosan films with *L. ferrea* require research to improve their structural properties and additional biological testing, such as cytotoxicity studies using different cell lines.

## 4. Materials and Methods

### 4.1. Collection of Plant Material

The stem bark of *L. ferrea* was collected in the Atlantic Forest biome in the city of Jussari—BA, Brazil (15°11′29″ S and 39°29′43″ W) on 11 December 2018 (spring, Jussari municipality, Bahia, Brazil). The specimen’s exsiccata was registered in the RADAMBRASIL Herbarium (HBS), Botanical Garden, Salvador-BA, under voucher specimen number 61850 [68].

### 4.2. Obtaining the Extract and Its Fractions

The ethanolic extract of the stem bark (ELF) was obtained by maceration in ethanol as described by Santana et al. (2024) [68]. To solubilize the extract, 10 g of the ethanolic extract of the stem bark of *L. ferrea* were transferred to a separating funnel and thoroughly mixed with 300 mL of a methanol: water solution [CH_3_OH:H_2_O (1:9)] (Methanol 99.8%, Química Moderna, Santana de Parnaíba, São Pualo, Brazil) until completely dissolved. This solution was submitted to liquid–liquid partitioning with ethyl acetate (150 mL, 3×) (Synth Labsynth, Diadema, São Paulo, Brazil), according to the methodology of OLIVEIRA et al. (2024) [93], with minor modifications. The organic fraction obtained was dried with anhydrous sodium sulfate (99.0% Synth Labsynth, Diadema, São Paulo, Brazil), and the solvent was removed by distillation under reduced pressure, in a rotary evaporator (Ika RV 10 Basic), yielding an ethyl acetate fraction, ELFA (5.96 g ± 0.28). The aqueous fraction, ELFAq, was subjected to freeze-drying (SL 404 from Solab Laboratory Equipment, Piracicaba, São Paulo, Brazil), yielding 3.40 g ± 0.20 g of this fraction.

### 4.3. Synthesis of Biofilms of Chitosan Incorporated with Samples of L. ferrea

Commercial chitosan (Polymar, Brazil, deacetylation degree of 87%) [123] was purified by crystallization with acetic acid (2% v v^−1^, 99.7% Dinâmica, Indaiatuba, São Paulo, Brazil) as described by KOC et al. (2020) [99]. Biofilms were synthesized according to Dos Santos (2024) [32], with modifications. The 100% chitosan film, without the addition of extract samples or fractions, was prepared by mixing 0.75 g of chitosan with 35 mL of acetic acid solution (of 2% v v^−1^), which was kept under stirring for 3 h in a magnetic stirrer (Fisatom Mod 752 A stirrer, Brazil). After this period, glycerol was added (0.0833 g, 10% v v^−1^) (glycerol, ACS Científica, Sumaré, São Paulo, Brazil). First, stir the mixture for another 21 h at room temperature. Next, gravity-filter the solution through Oxford cloth, then pour the filtered solution into a 20 cm-diameter polystyrene Petri dish. Finally, dry the solution at room temperature in a laminar-flow chamber (Vertical Unidirectional Flow 12—Veco Group, Campinas, São Paulo, Brazil). Similarly, chitosan films were prepared by incorporating the ethanol extract of *L. ferrea* stem bark and its ethyl acetate and water fractions, which were added after 23 h of stirring. Chitosan biofilms were prepared by incorporating 40% each respective of *L. ferrea* samples into the chitosan matrix. The following amounts were used to prepare each chitosan/*L. ferrea* biofilm: (a) Chitosan biofilm with ethanolic extract (BLFE): 0.45 g chitosan, 0.30 g extract; (b) Chitosan biofilm with aqueous fraction (BLFAq): 0.45 g chitosan, 0.30 g aqueous fraction; (c) Chitosan biofilm with ethyl acetate fraction (BLFA): 0.45 g chitosan, 0.30 g ethyl acetate fraction.

### 4.4. Antimicrobial Assay

Antimicrobial assays of *L. ferrea* extract and fractions were performed against the microorganisms *Staphylococcus aureus* (ATCC 6538), *Bacillus subtilis* (ATCC 6633), *Bacillus cereus* Frankland & Frankland (No. CCT 0198; ATCC 14579), *Escherichia coli* (ATCC 94863), *Pseudomonas aeruginosa* (No. CCT 0090; ATCC 27853), *Salmonella enterica subsp. enterica serovar Typhimurium* (CCT: 1478; ATCC 14028), *Candida albicans* (ATCC 18804). The microorganisms were obtained from the Tropical Culture Collection (CCT) of the André Tosello Foundation, Campinas, São Paulo, Brazil. They were reactivated and deactivated in the Bioassay Laboratory of the Department of Exact and Earth Sciences at the State University of Bahia I, Salvador, Bahia, Brazil.

#### 4.4.1. Microdilution Assay

The broth microdilution assay was used to determine the minimum inhibitory concentrations (MIC) of the ethanolic extract of *L. ferrea* stem bark (ELF), its aqueous and ethyl acetate fractions (ELFA and ELFAq), and pure chitosan, according to the Clinical and Laboratory Standards Institute (CLSI, 2020) [124] with modifications [68]. Only the experimental conditions will be described, as the microdilution assay methodology was identical to that reported in the literature [68]. The concentration of the stock solution in H_2_O-DMSO (20% *v*/*v*; DMSO: Synth 99.9%, São Paulo, Brazil) was 2000 µg mL^−1^ for the sample. In the experiment, the concentration range used for the sample was 500 to 3.9 µg mL^−1^.

The media used for the maintenance and cultivation of microorganisms were nutrient broth (NB) for bacteria (Acumedia, Lansing, MI, USA) and yeast and malt broth (YMB) for fungi (Acumedia, Brazil). Stock solutions, at a concentration of 200 µg mL^−1^, of tetracycline (Sigma-Aldrich, Chaoyang District, Beijing, China), gentamicin (Gibco, Pudong District, Shanghai, China), and ciclopirox olamine (EMS, Hortolância, São Paulo, Brazil) were used in experiments with Gram-positive bacteria, Gram-negative bacteria, and fungi, respectively. The initial inoculum for the experiment was turbidity at 0.5 McFarland scale. Bacteria and fungi were incubated in a BOD incubator (Bacteria: BOD incubator Quimis; Q315M, Diadema, São Paulo, Brazil). Fungi: Incubator Tecnal; TE371, Piracicaba, São Paulo, Brazil) at 37 °C (24 h), and fungi at 26 °C (72 h). After this period, the minimum inhibitory concentration (MIC) was visually determined. The minimum bactericidal concentration (MBC) and minimum fungicidal concentration (MFC) values were obtained as described in the literature [68].

#### 4.4.2. Agar Diffusion Assay

The antimicrobial activity of chitosan biofilms, both without (BCH100, pure chitosan film) and with the incorporation of the extract and its fractions (BLFE, BLFAq, and BLFA), as well as that of the extract and their fractions, was determined using the agar diffusion method. This followed the procedure described by DOS SANTOS et al. (2024) [32]. 20 mL of nutrient agar (NA) culture medium (Acumedia, Lansing, MI, USA) was transferred to a Petri dish (9 × 10 cm). After the medium solidified, 300 µL of the microorganism inoculum [turbidity 0.5 on the McFarland scale (~0.5 × 10^8^ CFU/mL)] was added, and the inoculum was uniformly spread using a Drigalski loop. Immediately after seeding, wells with a 6 mm diameter were drilled into the medium. In these wells, 20 µL of the stock solutions of the extract, fractions, and chitosan (8000 µg mL^−1^) and the antibiotics tetracycline (10 µg mL^−1^), gentamicin (400 µg mL^−1^), and ciclopirox olamine (200 µg mL^−1^) were added. To accommodate the agar diffusion assay with biofilms, some adaptations were made. Specifically, the biofilms were cut into discs (6 mm in diameter) and transferred to the solid medium inoculated with the microorganism. Following this, all plates were properly sealed and incubated (bacteria for 24 h at 37 °C and fungi for 72 h at 26 °C) in a BOD incubator (Bacteria: Quimis; Q315M. Fungi: Tecnal; TE371). After the incubation period, the inhibition zones were measured with a millimeter ruler. The entire experiment was conducted in triplicate, and standard deviation values were obtained.

### 4.5. Determination of Total Phenolics

The determination of the total phenolic of the ethanolic extract of *L. ferrea* stem bark and its fractions in ethyl acetate and water was carried out using Folin–Ciocalteu spectrophotometric method, according to the literature [32,68]. The reagents and solvents used in these methods were methanol (99.8%, Química Moderna, Santana de Parnaíba, São Pualo, Brazil), Folin–Ciocalteu reagent (Merck, Darmstadt, Germany), sodium carbonate (99.5%, Synth Labsynth, Diadema, São Paulo, Brazil); gallic acid (Vetec Sigma Aldrich 98%, Duque de Caxias, Rio de Janeiro, Brazil). Total Phenolics: a 20 µL aliquot of a methanolic solution of the sample (extract or fraction) (1 mg mL^−1^) was transferred to wells of a 96-well microplate. To these wells, 20 µL of aqueous Folin–Ciocalteu reagent solution (10% v v^−1^), 60 µL of aqueous sodium carbonate solution (7.5% m v^−1^), and 200 µL of distilled water were added. The microplate was then incubated for 20 min at room temperature, protected from light. After incubation, readings were taken at 760 nm using a UV/VIS spectrophotometer (LMR 96, Loccus, Cotia, São Paulo, Brazil). The calibration curve was constructed similarly with gallic acid at concentrations of 1, 2, 4, 6, 8, 10, 15, 20, 40, and 60 µg mL^−1^.

### 4.6. Sample Characterization

#### 4.6.1. Nuclear Magnetic Resonance Analysis

The aqueous and ethyl acetate fractions, obtained from the ethanolic extract of the stem bark of *L. ferrea*, were subjected to Nuclear Magnetic Resonance analysis. The equipment used was the Bruker AVANCE III 9.4 Tesla (400 MHz) from the Nuclear Magnetic Resonance Laboratory of the Organic Chemistry Department at the Federal University of São Carlos (UFSCar). The samples were solubilized in deuterated methanol.

#### 4.6.2. Fourier Transform Infrared Spectroscopy (FTIR) Analysis

FTIR data from extract samples, fractions, chitosan, and chitosan biofilms with and without the incorporation of *L. ferrea* samples were acquired on a Fourier Transform Infrared Spectrometer (FTIR) (Perkin Elmer Spectrum Two FTIR model), in the range of 4000–450 cm^−1^ (resolution of 4 cm^−1^), in attenuated total radiation (ATR) mode, with an accumulation of 8 scans.

#### 4.6.3. Thermal Properties of Chitosan Biofilms and Chitosan/*L. ferrea* Biofilms

Thermogravimetric data for the extract, *L. ferrea* fractions, chitosan, and chitosan-based biofilms were collected using a DTG-60H Analyzer (SHIMADZU, Nishinokyo-Kuwabara-cho, Kyoto, Japão). Samples were heated from 25 to 800 °C under nitrogen at 50 mL min^−1^ and 10 °C min^−1^, in an aluminum crucible.

#### 4.6.4. Microstructure Analysis of Chitosan Biofilms and Chitosan/*L. ferrea* Biofilms

Morphological analyses of chitosan biofilms with and without the incorporation of *L. ferrea* were performed using a Scanning Electron Microscope (JSM-6610LV, Jeol, Oxford Instruments, Pleasanton, CA, USA). The samples were covered with a thin layer of gold (Denton Vacuum, Desk V). The operating conditions were: accelerometer voltage: 10KV; sensor/sample distance: WD10 mm; magnification: ×80, ×300, and ×1800. Energy-dispersive spectroscopy (EDS) was also performed at x1000 magnification to determine the composition of the samples’ surface layers, which were processed using AZtec software (AZtec 3.3 SP1).

#### 4.6.5. Thickness of Chitosan Biofilms and Chitosan/*L. ferrea* Biofilms

Biofilm thickness, with and without the *L. ferrea* sample, was measured using a digital micrometer (Aquibras). Ten measurements from different regions were taken for each biofilm, and their arithmetic mean, reported in millimeters (mm), was calculated.

#### 4.6.6. Mechanical Properties of Chitosan Biofilms and Chitosan/*L. ferrea* Biofilms

Mechanical testing of chitosan biofilms, with and without ethanolic extract and *L. ferrea* fractions, was conducted using a scanning electron microscope (EMIC, DL-200 MF, Brazil). Ten specimens were used to analyze rupture strength (N) and stress (MPa). Test parameters were: speed of 500 mm/min, return speed of 100 mm/min, tensile strength limit of 100 N, and strain limit of 200 mm.

### 4.7. Biodegradability of Chitosan Biofilms and Chitosan/L. ferrea Biofilms

The soil biodegradation assay was conducted according to Oberlintner et al. (2020) [110] and De Carli et al. (2022) [125], with a few modifications. The soil used in the experiment was collected on campus I of the State University of Bahia (−12.951944, −38.459714). The biofilm samples were cut into fragments (2 cm × 3 cm) and buried in the soil, approximately 5 cm deep, in polystyrene vessels. The initial masses of the biofilms (Mi) were measured before immersion in soil. The samples were then incubated at 26 ± 1 °C for 25 days in a climate-controlled chamber, with 10 mL of water added on alternate days to moisten the soil. After incubation, biofilms were cleaned with a soft brush, rinsed with distilled water, dried at room temperature, and their final masses (Mf) were recorded. The experiment was conducted in triplicate.

The biofilm biodegradation assay in water was continuous [125,126]. First, the initial masses (Mi) of biofilm fragments (2 cm × 3 cm) were obtained. Next, these fragments were immersed in 30 mL of water and incubated in a climate-controlled chamber at 26 ± 1 °C. After 25 days of incubation, the samples were removed from the aqueous medium. Then, surface water was removed with filter paper. Finally, the samples were placed in an oven at 50 °C to dry and determine their final masses (Mf). The percentage of biodegradation in soil and water was calculated gravimetrically using the equation. Biodegradation = [(Mi − Mf)/Mi] × 100 [125,127].

## 5. Conclusions

This study reinforces the antibacterial potential of chitosan biopolymers incorporating extracts and fractions of *L. ferrea*, a medicinal plant. To advance this research, further studies are needed to purify and structurally identify the main secondary metabolites of the ethanolic extract from the stem bark. Additionally, it is necessary to improve the mechanical properties of the biofilms by incorporating the extract and fraction ethyl acetate without affecting their antibacterial activities. However, the chitosan film incorporating the aqueous fraction showed the greatest potential for biomedical applications. Together, these efforts will enable their long-term application as a dressing or antibacterial packaging.

## Figures and Tables

**Table 1:** Mass loss events of chitosan biofilm and chitosan biofilms incorporated into the extract and fractions of *L. ferrea*.

	ΔT_1_ (°C)	Δm_1_ (%)	Tp_1_ (°C)	ΔT_2_ (°C)	Δm_2_ (%)	Tp_2_ (°C)	ΔT_3_ (°C)	Δm_3_ (%)	Tp_3_ (°C)
BCH100	39.26–59.63	14.467	46.15	230.43–287.76	42.437	262.09	504.06–541.73	34.365	526.54
BLFE	37.78–63.79	14.500	45.68	220.20–296.67	35.152	255.19	510.84–593.26	40.770	552.43
BLFA	40.72–74.55	12.009	43.56	208.74–310.20	37.17	263.89	632.50–739.90	34.984	666.26
BLFAq	45.39–73.07	19.731	47.81	224.40–300.98	33.379	247.12	684.26–780.0	41.467	759.25

ΔT: Temperature range at each event; Δm: percentage change in mass at each event; Tp: Peak temperature of the DTG at each event. BCH100 (pure chitosan biofilm), BLFE (chitosan biofilm incorporating ethanolic extract of *L. ferrea* stem bark), BLFA (chitosan biofilm with ethyl acetate fraction), and BLFAq (Chitosan biofilm with aqueous fraction).

**Table 2:** Thickness and mechanical parameters of biofilms with and without incorporation of *L. ferrea* samples.

Sample	Thickness (mm)	Rupture (N)	Tensão (Mpa)
BCH100	0.021± 0.002	17.50 ± 14.81	28.62 ± 15.31
BLFE	0.032 ± 0.002	39.83 ± 13.22	52.78 ± 18.50
BLFA	0.019 ± 0.001	40.05 ± 19.81	87.43 ± 41.98
BLFAq	0.029 ± 0.005	40.92 ± 11.30	60.58 ± 19.53

BCH100: Pure chitosan biofilm; BLFE: chitosan biofilm incorporating ethanolic extract of *L. ferrea* stem bark; BLFA: chitosan biofilm with ethyl acetate fraction; BLFAq: chitosan biofilm with aqueous fraction.

**Table 3:** Biodegradation of biofilms in water and soil.

	Weight Loss (%)	
Biofilms	Soil	Water
BCH100	47.8 ± 4.2	42.3 ± 6.5
BLFAq	36.2 ± 3.8	29.8 ± 4.1
BLFA	34.1 ± 5.9	28.7 ± 7.2

BCH100: pure chitosan biofilm; BLFA: chitosan biofilm with ethyl acetate fraction of ethanolic extract of *L. ferrea* stem bark; BLFAq: chitosan biofilm with aqueous fraction of ethanolic extract of *L. ferrea* stem bark.

**Table 4:** Antibacterial activity by the agar diffusion method.

Inhibition Zones (IZ, mm)					
Sample	*S. aureus*	*B. cereus*	*B. subtilis*	*E. coli*	*P. aeruginosa*
BLFA	12.0 ± 0.1	10.0 ± 0.1	-	-	-
BLFAq	12.0 ± 0.1	10.0 ± 0.1	-	-	-
BLFE	14.1 ± 0.1	-	-	-	-
BCH100	-	13.6 ± 0.1	-	-	-
ELF	14.0 ± 0.1	14.0 ± 0.1	-	-	-
ELFA	12.3 ± 0.1	10.3 ± 0.2	-	-	-
ELFAq	12.8 ± 0.2	10.8 ± 0.1	-	-	-
Tetracycline	33.0 ± 0.1	32.0 ± 0.1	33.0 ± 0.1		
Gentamicin				14.0 ± 0.1	15.0 ± 0.1

-: There was no IZ; BCH100: pure chitosan biofilm; BLFE: chitosan biofilm incorporating ethanolic extract of *L. ferrea* stem bark; BLFA: chitosan biofilm with ethyl acetate fraction; BLFAq: chitosan biofilm with aqueous fraction. ELF: ethanolic extract of stem bark, ELFAq: aqueous fraction, and ELFA and ethyl acetate fraction. Tetracycline (10 µg mL^−1^), Gentamicin (400 µg mL^−1^), and extracts and fractions (8000 µg mL^−1^).

**Table 5:** Antibacterial activity by broth microdilution assay of aqueous and ethyl acetate fractions of the ethanolic extract of *L. ferrea* stem bark.

Samples	*S. aureus*		*B. cereus*		*B. subtilis*		*P. aeruginosa*		*E. coli*	
	MIC and MBC Values in µg mL^−1^									
	MIC	MBC	MIC	MBC	MIC	MBC	MIC	MBC	MIC	MBC
ELFAq	250	500	250	250	>500	-	250	>500	>500	-
ELFA	125	500	125	125	>500	-	250	>500	>500	-
Tetracycline	7.8	7.8	7.8	7.8	7.8	7.8				
Gentamicin							15.6	62.5	0.3	0.3

˃: no >: inhibitory effect on growth at the highest concentration tested [500 µg mL^−1^]. ELFAq: aqueous fraction of the ethanolic extract of the stem bark of *L. ferrea*, and ELFA and ethyl acetate fraction of the ethanolic extract of the stem bark of *L. ferrea*: Tetracycline (100 µg mL^−1^) and gentamicin (200 µg mL^−1^). Ethyl acetate fraction of the ethanolic extract of the stem bark of *L. ferrea*.

## Data Availability

The data presented in the manuscript can be requested from the authors via email.

## Supplementary Materials

The following supporting information can be downloaded at: https://www.mdpi.com/article/10.3390/molecules31091392/s1. Figure S1: 1H NMR spectra (CD3OD, 400 MHz), (a) Ethanolic extract of L. ferrea stem bark (ELFA); (b) Ethyl acetate fractions of the ethanolic extract (ELFA); and (c) Aqueous fraction of the ethanolic extract (ELFAq); Figure S2: Energy dispersive spectroscopy spectra of biofilms.
