# Supplementary material for: Antibacterial Biofilms of Chitosan Incorporated with the Ethanolic Extract of the Stem Bark of Libidibia ferrea and Its Fractions
Source: Molecules. 2026 Apr 23;31(9):1392. doi: 10.3390/molecules31091392 (PMC13165208; doi:10.3390/molecules31091392)
Supplement: Supplementary file 1 [file molecules-31-01392-s001.zip › molecules-4215448-supplementary.pdf]

SUPPLEMENTARY DATA

Antibacterial Biofilms of Chitosan Incorporated with the Ethanolic Extract of the Stem Bark of *Libidibia ferrea* and its Fractions

Andreza Santos de Jesus[1], Aiane Nascimento Santana[1], Helena Carla Magalhães dos Reis[1], Giovanna Regina Gonzalez de Santana Wojnar[1], Vitor Hugo Migues[1], Arnaud Victor dos Santos[1], Madson de Godoi Pereira[1], Lourdes Cardoso de Souza Neta[1,*], Sandra Aparecida Alexandre Lucas[2] and Rodrigo Lassarote Lavall[2]

[1] Department of Exact and Earth Sciences I, UNEB-State University of Bahia, Silveira Martins Street, 2555 Cabula, 41150-000 Salvador, Bahia, Brazil; dreza.santos@hotmail.com (ASJ); aianesantana92@gmail.com (ANS); Hcarla01.HC@gmail.com (HCMR); vhmigues@gmail.com (VHM); arnaudvic@gmail.com (AVS); mpereira@uneb.br (MGP); lcsneta2@gmail.com (LCSN)

[2] Department of Chemistry, Institute of Exact Sciences, UFMG-Federal University of Minas Gerais, Antônio Carlos Av., 6627 Pampulha, 31270-901 Belo Horizonte, Minas Gerais, Brazil; salexandre2007@gmil.com (SAAL); rodrigo.lavall@qui.ufmg.br (R.L.L.)

* Correspondence: lcsneta2@gmail.com (or lcneta@uneb.br)

a)

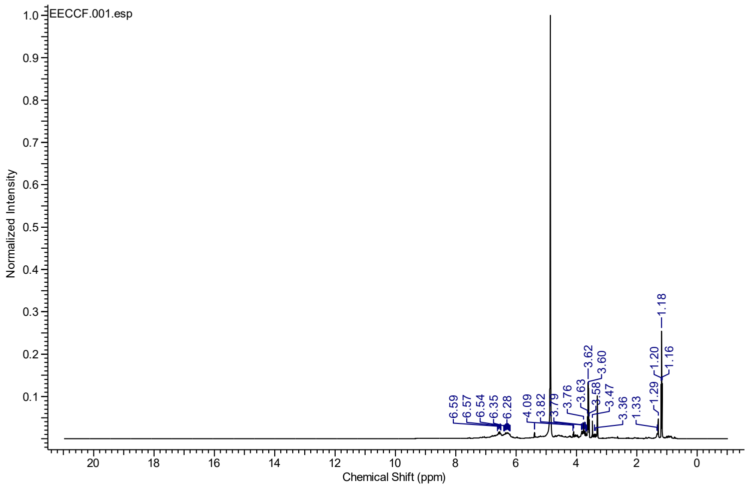

b)

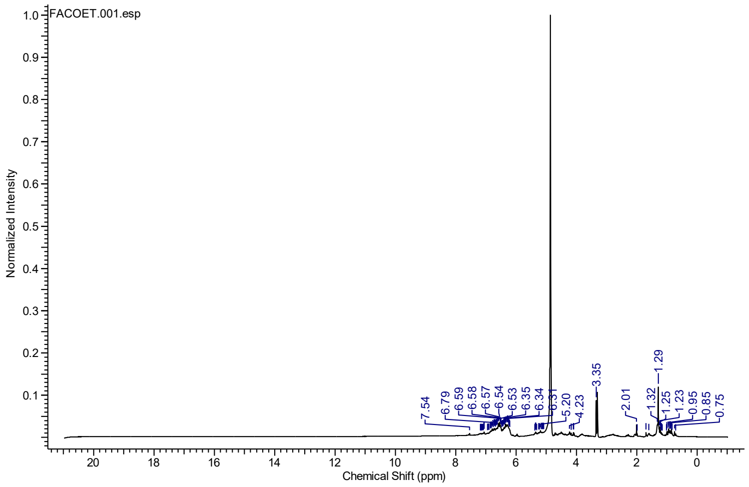

c)

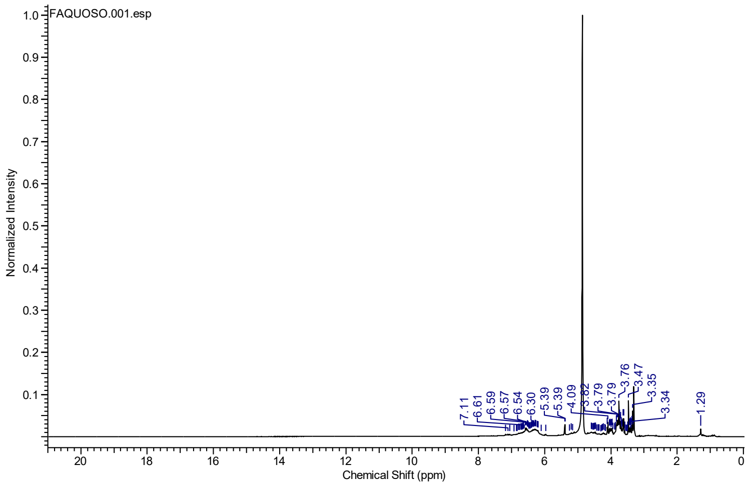

Figure S1. ^{1}H NMR spectra (CD_3OD, 400 MHz), a) Ethanolic extract of *L. ferrea* stem bark (ELFA); b) Ethyl acetate fractions of the ethanolic extract (ELFA); and c) Aqueous fraction of the ethanolic extract (ELFAq).

a) BCH100

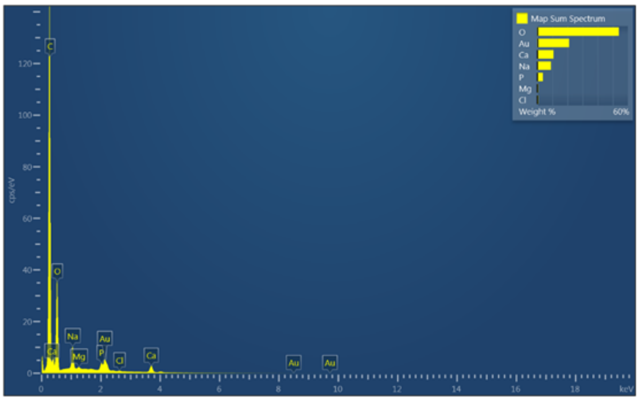

b) BLFA

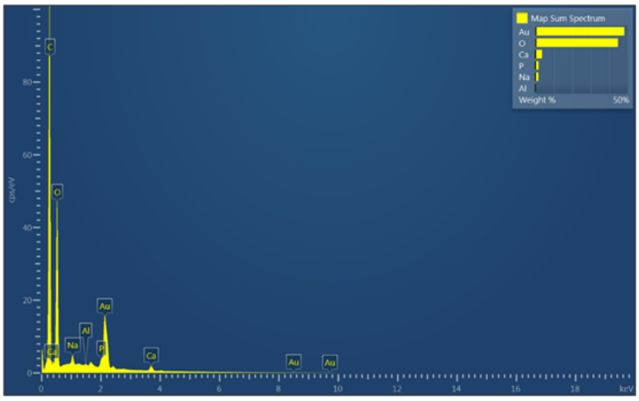

c) BLFAq

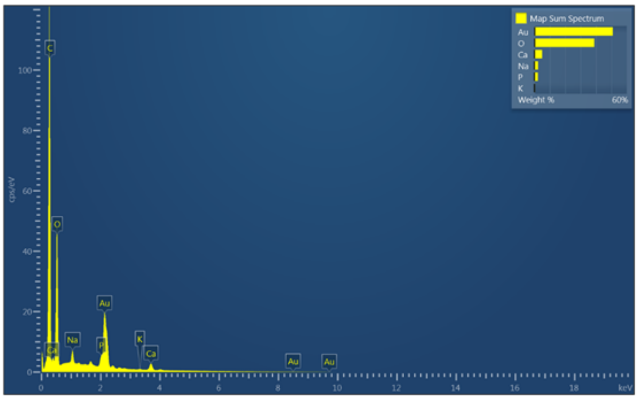

Figure S2. Energy dispersive spectroscopy spectra of biofilms.

*BCH100: pure chitosan biofilm; BLFE: chitosan biofilm incorporating ethanolic extract of *L. ferrea* stem bark; BLFA: chitosan biofilm with ethyl acetate fraction; BLFAq: chitosan biofilm with aqueous fraction.

.
